# Comprehensive analysis of the PD-L1 and immune infiltrates of N6-methyladenosine related long non-coding RNAs in bladder cancer

**DOI:** 10.1038/s41598-022-14097-x

**Published:** 2022-06-16

**Authors:** M. Q. Xue, Y. L. Wang, J. C. Wang, X. D. Wang, X. J. Wang, Y. Q. Zhang

**Affiliations:** 1 The First Affiliated Hospital of Zhengzhou University, Zhengzhou, 450052 People’s Republic of China; 2 Henan Bioengineering Technology Reseach Center, Zhengzhou, 450010 People’s Republic of China; 3 Zhengzhou Technical College, Zhengzhou, 450010 People’s Republic of China; 4 Henan General Hospital, Zhengzhou, 450002 People’s Republic of China

**Keywords:** Cancer, Computational biology and bioinformatics

## Abstract

Bladder cancer (BLCA) is one of the most frequent genitourinary cancers, with a high rate of morbidity and mortality. The connection of m6A-related lncRNAs with PD-L1 and tumor immune microenvironment (TIME) in BLCA prognosis was extensively investigated in this study, which could suggest novel therapeutic targets for further investigation. 30 m6A-associated lncRNAs with predictive values from the TCGA data set were identified with co-expression analysis. Cluster2 was correlated with a poor prognosis, upregulated PD-L1 expression, and higher immune ratings. Cluster2 had larger amounts of resting CD4 memory-activated T cells, M2 macrophages, neutrophils, and NK cells infiltration. “CHEMOKINE SIGNALING PATHWAY” was the most significantly enriched signaling pathway according to GSEA, which may play an important role in the different immune cell infiltrates between cluster1/2. The risk model for m6A-related lncRNAs could be employed in a prognostic model to predict BLCA prognosis, regardless of other clinical features. Collectively, m6A-related lncRNAs were linked to PD-L1 and TIME, which would dynamically affect the number of tumor-infiltrating immune cells. m6A-related lncRNAs may be key mediators of PD-L1 expression and immune cells infiltration and may strongly affect the TIME of BLCA.

## Introduction

Bladder cancer is the ninth most frequent malignancy in the world. It is mainly represented by bladder urothelial cancer (BUC), accounting for more than 90% of bladder cancer, and smoking is considered the leading risk factor^1,2^. Furthermore, BLCA has a significant recurrence rate, and approximately half of patients relapsing following major surgery and developing metastases^3,4^. Patients with metastatic urothelial carcinoma have reaped extraordinary gains from platinum-based chemotherapy and Immune Checkpoint Inhibitors (ICIs), but the diverse features of BLCA prompt to variable clinical outcomes for BLCA patients even though they received standard therapy^5^. Bladder cancer develops through a complex process including aberrant genetic changes and epigenetic aberrations. Epigenetic anomalies can be seen on a range of levels, including DNA^6^, RNA^7^ and histone modifications^8–10^. Researchers must find novel biomarkers to better predict the prognosis and therapeutic response to BLCA in order to enhance survival and minimize the burden of BLCA patients.

Long noncoding RNAs (lncRNAs) are a class of noncoding RNA with a length of more than 200 nucleotides that have been implicated in post-transcriptional regulatory elements that regulate mRNA splicing, stability, and translation^11,12^. LncRNAs may also play a key role in regulating genes that code for cancer-fighting proteins, according to growing evidence^13^. NKILA lncRNA, for example, aids tumor immune evasion by making T cells more susceptible to activation-induced cell death^14^. By generating an RNA–protein complex with enhancer of zeste homolog2(EZH2) and boosting the binding of EZH2 and H3K27me3 on the E-cadherin promoter region, lncRNA MRPL23-AS1 facilitated adenoid cystic carcinoma lung metastasis^15^. However, the potential role of immune-related lncRNA signatures as an effective therapeutic strategy in BLCA is unknown.

N6-methyladenosine (m6A) alteration has been shown to regulate lncRNAs in several research^16–19^. M6A, the methylation modification at the sixth N atom of adenine, is the most frequent post-transcriptional alteration on mRNA, mediating > 60% RNA methylation^20,21^. The m6A enzyme’s abnormal expression affects tumor cell function and the tumor microenvironment (TIME)^22–24^. Tumor stem cell self-renewal is accelerated by abrupt m6A alteration, which plays a vital role in tumorigenesis^25^. m6A enzymes and lncRNAs are both excellent diagnostic and prognostic indicators. Evidence is overwhelming indicating m6A-related mRNAs and lncRNAs could be served as promising targets for estimating prognosis in a multitude of malignancies^26–29^. Degradation of m6A reader YTHDF2-modified lncRNA FENDER, for example, enhanced endometrioid endometrial tumorigenesis dramatically^30^. Wang et al.^31^ discovered that m6A-induced lncRNA RP11 expression triggers the malignant and immunosuppressive gastric cancer cells via upregulation of YAP1 expression. Therefore, Identification of novel and reliable prognostic molecular characteristics from various dimensions is critical for identifying the suitable therapy methods and improving the dismal prognosis in BLCA patients.

As tumors develop, the immune system is triggered to resist tumor development^32^. Several immunological checkpoints, such as programmed death ligand-1 (PD-L1), have been identified as one of the immune escape mechanisms^33^. The TIME may influence a patient’s response to immune checkpoint inhibitors, hence PD-L1 expression in BLCA should be taken into account while analyzing tumor immunity^34^. This study focus on systematically assessing the relationships of m6A-related lncRNAs with prognosis, programmed death ligand 1 (PD-L1), and TIME in BLCA.

## Results

### Identification of m6A related lncRNAs

**Figure 1:**
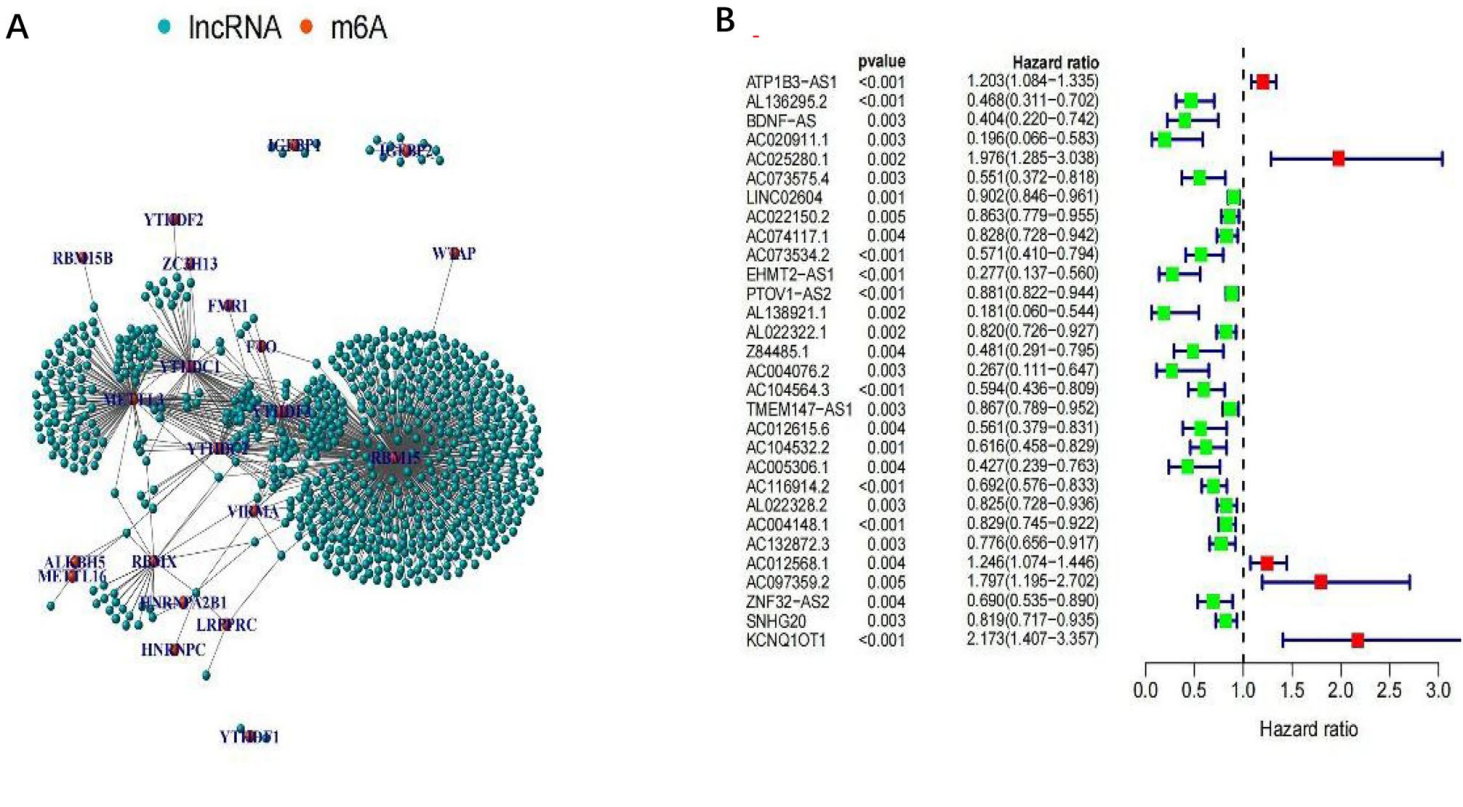
The expression of m6A-long noncoding RNAs (lncRNAs) and their function in the prognosis of BLCA patients. (**A**) Network plot of correlation between m6A related gene expression and lncRNAs. (**B**) forest plot of univariate cox regression analysis. The confidence interval and hazard ratio were determined with data from prognostic associated lncRNAs. Red represents high risk, while green represents low risk.

**Figure 2:**
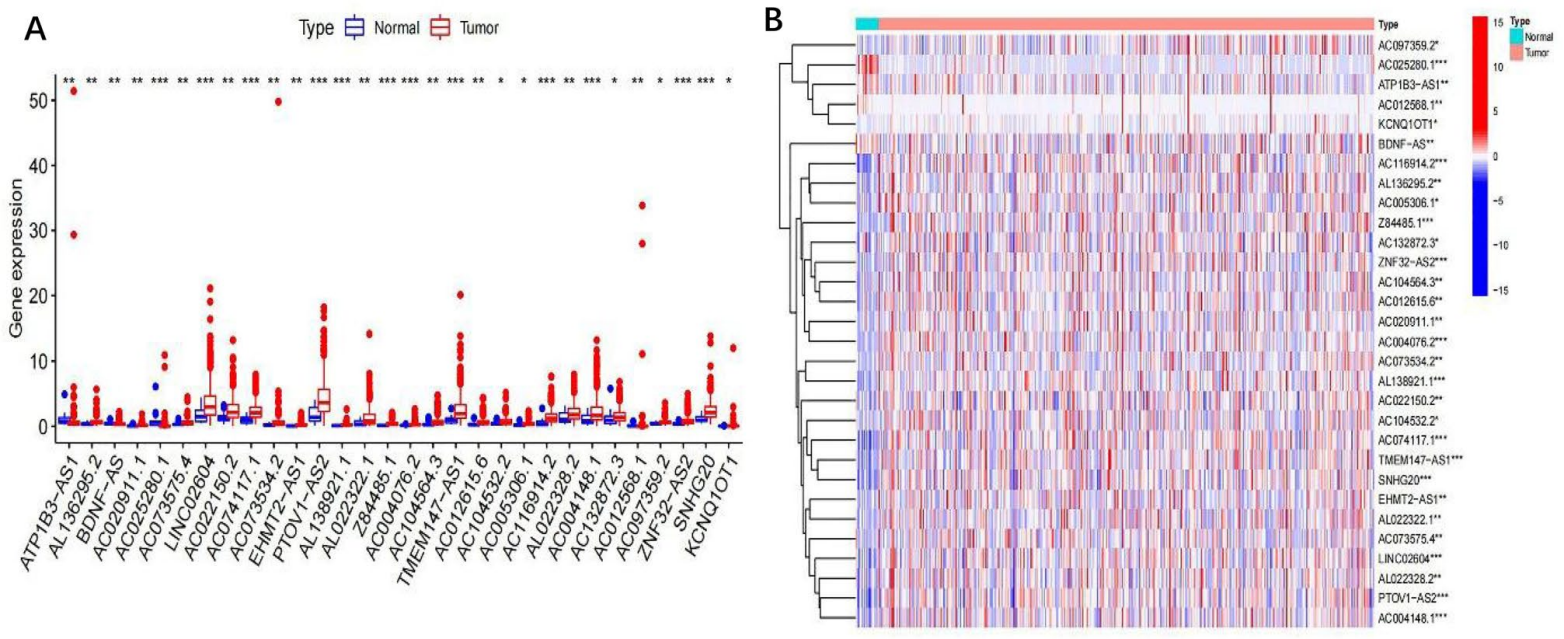
Different expression of m6A prognostic related lncRNAs. (**A**) Boxplot of the difference expression of m6A prognostic related lncRNAs among tumor and normal tissue. *p < 0.05; **p < 0.01; ***p < 0.001. (**B**) Heatmap of the difference expression of m6A prognostic related lncRNAs among tumor and normal tissue. *p < 0.05; **p < 0.01; ***p < 0.001. Red represents high expression, while blue represents low expression. The abscissa represents the sample, while the ordinate represents prognostic related lncRNA.

To distinguish mRNAs and lncRNAs, transcriptome data on m6A-related gene expression were retrieved from TCGA. Next, the connection between m6A-related gene expression and lncRNAs was analyzed via co-expression analysis (Table S1). A network plot was generated to visualize this correlation (Fig. 1A). A forest plot was used to visualize the univariate Cox regression analysis (Fig. 1B, Table S2). Heatmaps and box plots were constructed using the differential expression of prognosis-related m6A-related lncRNAs between tumor and normal tissues, as shown in Fig. 2A,B. 30 m6A prognosis-related lncRNAs were identified that were differently expressed between tumor and surrounding normal tissues. LINC02604, AC116914.2, Z84485.1, ZNF32–AS2, AC004076.2, AL138921.1, TMEM147–AS1, SNHG20, PTOV1–AS2 and AC004148.1 were dramatically higher in BLCA tissues than in normal adjacent tissues (p < 0.001). The expression level of ATP1B3–AS1, AC025280.1, AC012568.1, BDNF–AS was markedly lower in BLCA tissues than in normal tissues (p < 0.01).

### The role of m6A-related lncRNAs

**Figure 3:**
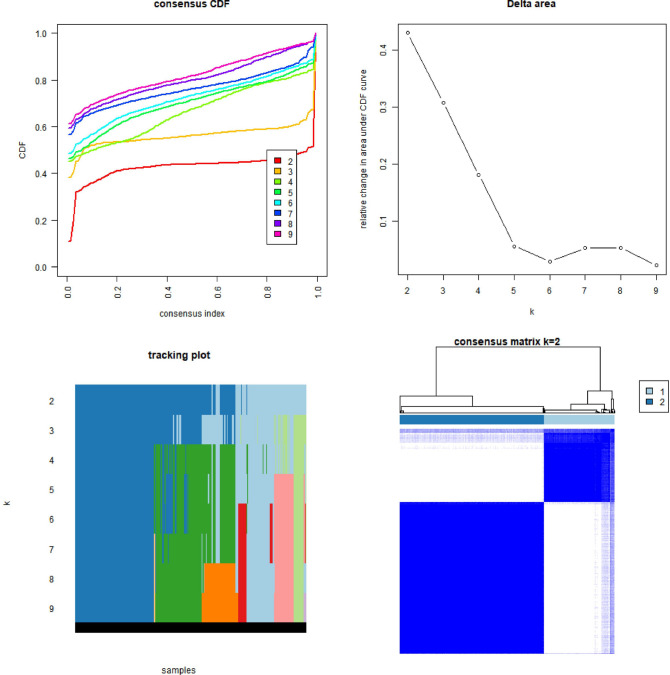
The type of prognostic m6A-related lncRNAs. We divided lncRNAs into two categories according to their expression: cluster1 and cluster2. When K = 2, there was the least cross-mixing part between the two types and the CDF value was lowest, thus we classified them into two types: cluster1 and cluster2.

**Figure 4:**
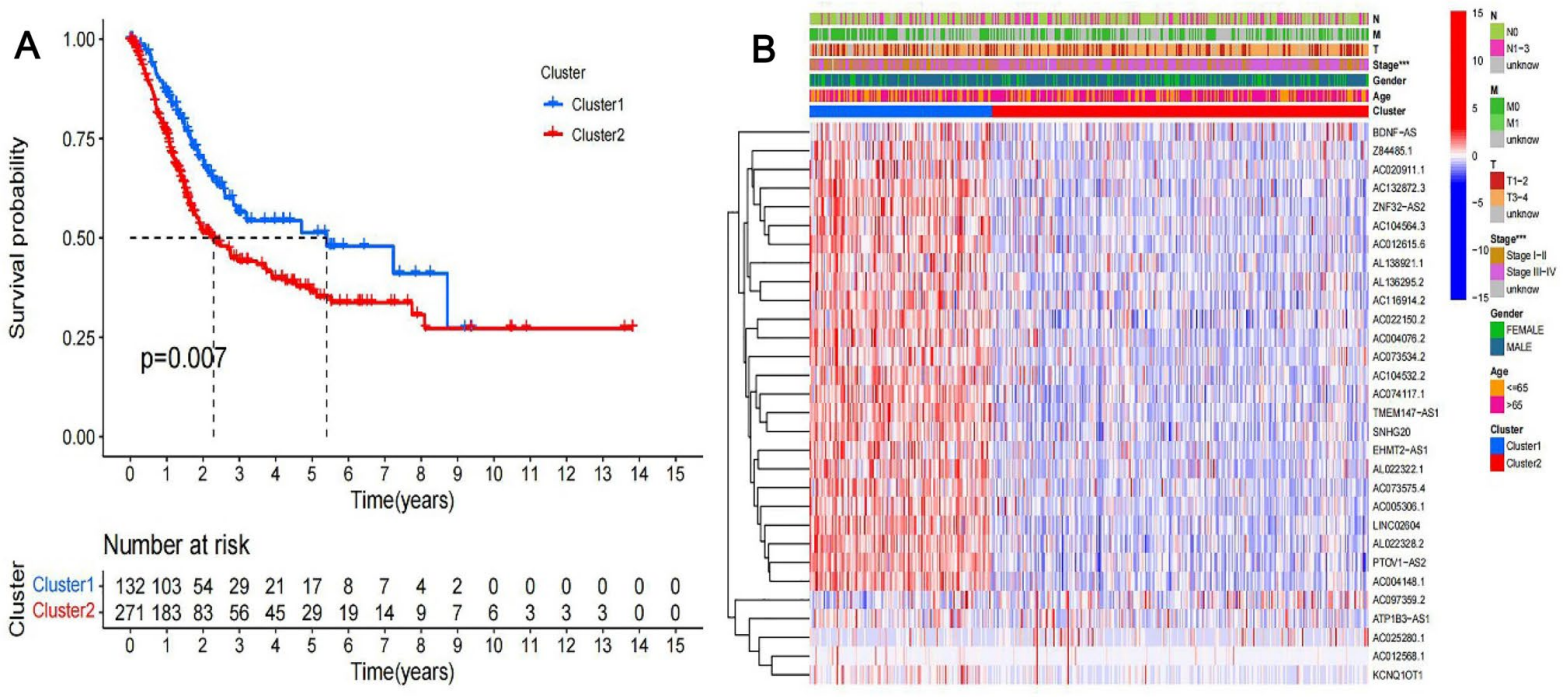
Prognostic associated m6A lncRNAs survival analyses and correlation with clinicopathological factors. (**A**) Survival analysis according to subtypes of lncRNAs, the survival rate of cluster1 was higher, p = 0.007. (**B**) Heatmap of difference expression of prognostic related lncRNAs and relationship with clinicopathological parameters in different cluster. *p < 0.05, **p < 0.01, and ***p < 0.001. Red represents high expression, while blue represents low expression. The abscissa represents the sample, while the ordinate represents prognostic related lncRNA.

Based on the similarity demonstrated by the expression levels of m6A-related lncRNAs and the proportion of ambiguous clustering measure, the k = 2 was identified as having optimal clustering stability from k = 2 to 9. A total of 433 patients with BLCA were clustered into two subtypes, namely, cluster1 (n = 132) and cluster2 (n = 271), based on the expression levels of the m6A-related lncRNAs (Fig. 3, Table S3). To assess the potential role of m6A-related lncRNAs, a survival study based on lncRNA subtypes was conducted, and the overall survival (OS) of cluster1 was higher than that of cluster2 (p = 0.007), as shown in Fig. 4A. The expression of most m6A-related lncRNAs was lower in the cluster2 than in the cluster1, especially the expression levels of LINC02604, AC104532.2, AL022328.2 and EHMT2–AS1 (Fig. 4B). While the expression of individual m6A-related lncRNAs was higher in the cluster2 than in the cluster1, such as AC097359.2 and AC025280.1 (Fig. 4B). Additionally, The clinicopathological features between the two subtypes were then compared (Fig. 4B). The cluster2 was preferentially associated with Stage III–IV (p < 0.001).

### Association of PD-L1 with m6A-related lncRNAs

**Figure 5:**
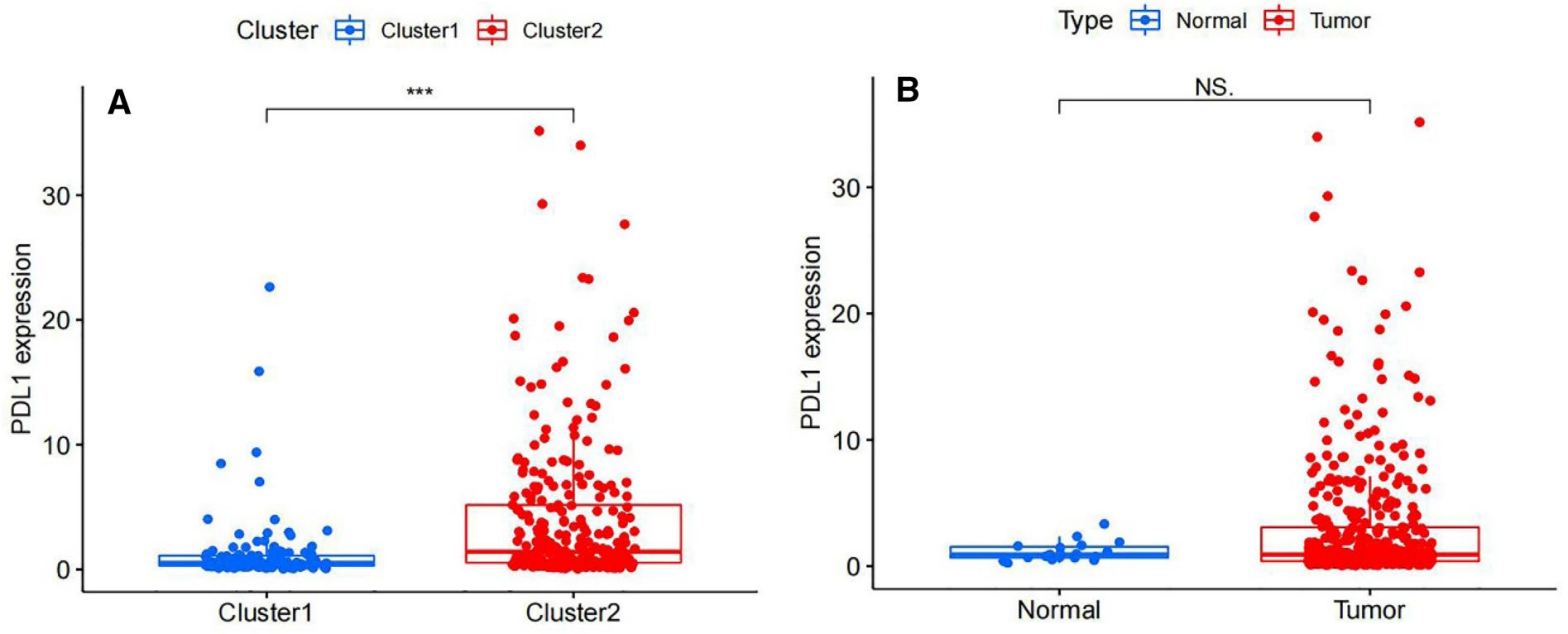
(**A**) The expression level of PD-L1 was upregulated in the cluster2 compared with cluster1 (p <  0.001). (**B**) Different expression of PD-L1 in related tissues. There was not significant difference between BLCA tissue and normal tissue (p > 0.05).

**Figure 6:**
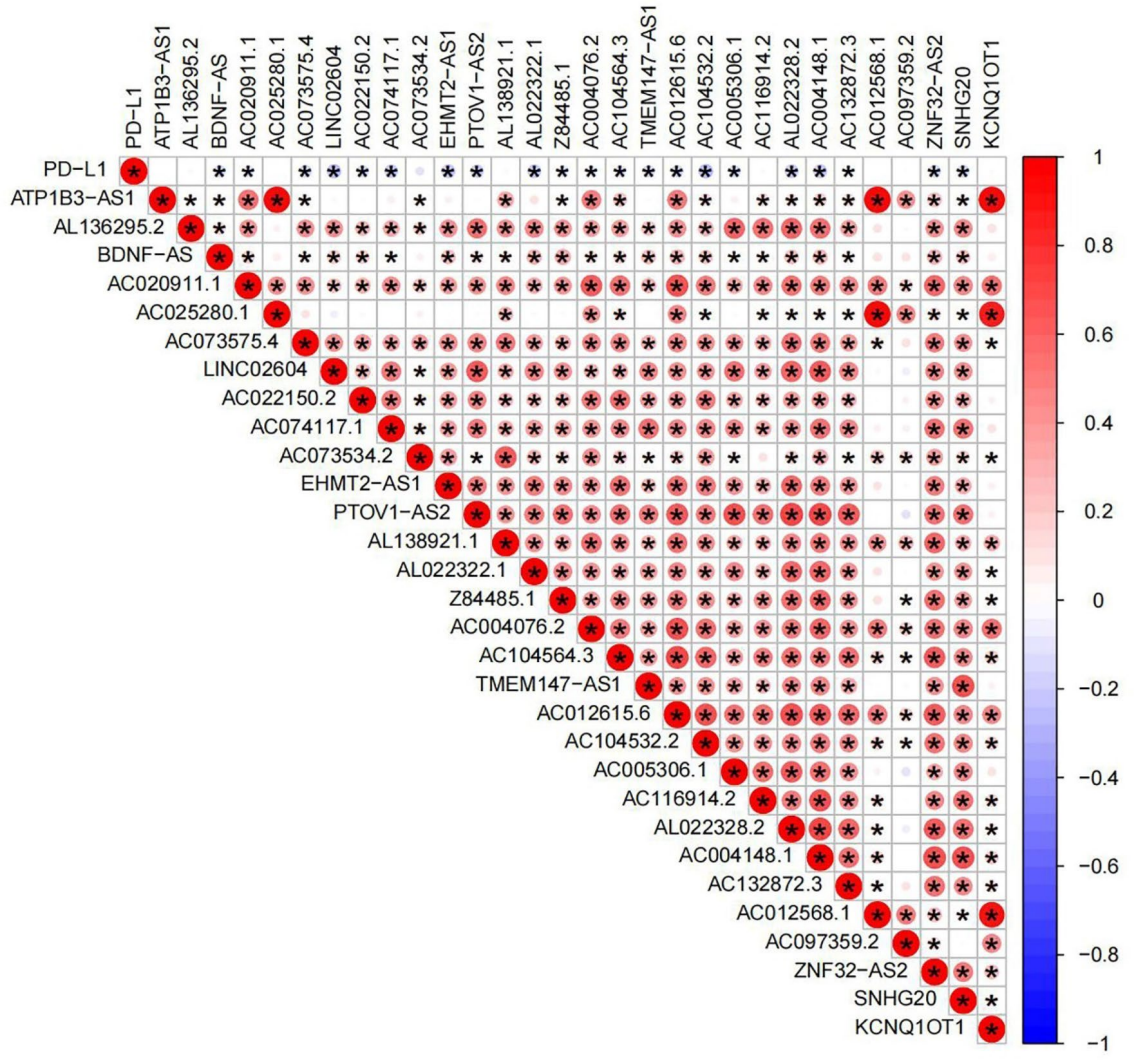
Association of PD-L1 with m6A-related lncRNAs. Red means positive correlation, while blue means negative correlation, *means the difference is statistically meaningful (p < 0.05).

To explore the correlation of PD-L1 with m6A-related lncRNAs, the differential expression of PD-L1 in two subtypes were assessed. The expression level of PD-L1 in the cluster2 was distinctly higher than that in the cluster1 (p < 0.01; Fig. 5A). However, there was no distinct difference in the expression of prognosis-related lncRNAs in the different tissues (Fig. 5B), probably due to small sample size. In BLCA, a gene correlation study was performed to see if there was a link between the target gene and the prognostic m6A-related lncRNAs (Fig. 6), and we found that the expression of PD-L1 had a significantly negative association with m6A-lncRNA LINC02604, AC104532.2, AL022328.2 and EHMT2–AS1.

### The role of immune cells infiltration and the tumor microenvironment

**Figure 7:**
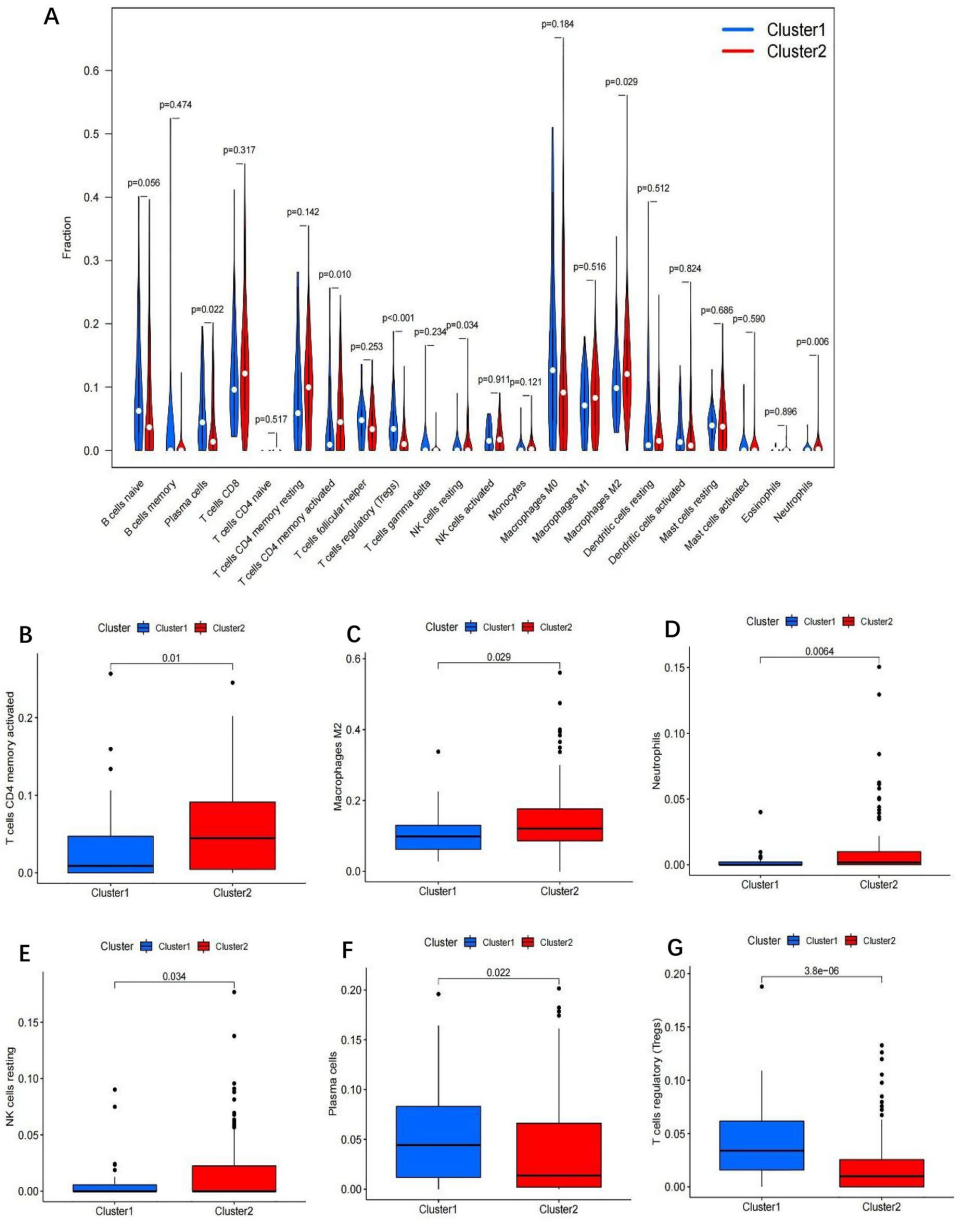
Different analysis of immune cell infiltration in different cluster. Vioplot: (**A**); Boxplot: (**B**); T cells CD4 memory activated, (**C**) macrophages M2, (**D**) Neutrophils, (**E**) NK cells resting, (**F**) plasma cells and (**G**) T cells regulatory (Tregs).

**Figure 8:**
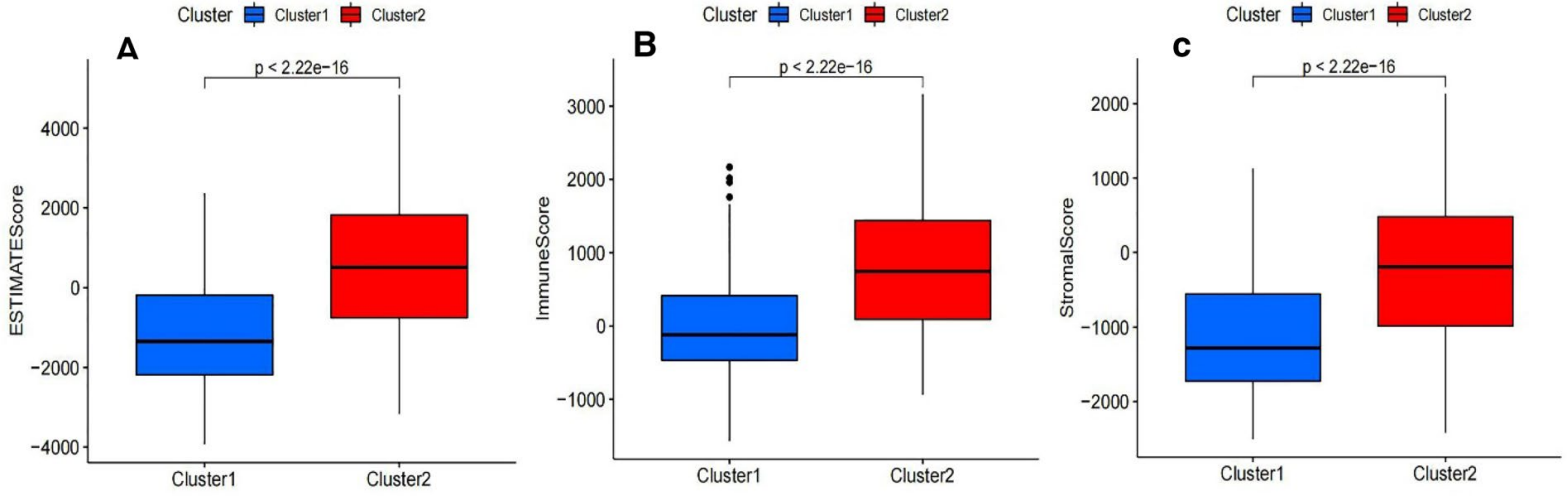
Immune scores in the cluster1/2 subtypes. (**A**) ImmuneScore, (**B**) ESTIMATEScore, (**C**) StromalScore. All of the scores are higher in cluster1 (p < 0.001).

**Figure 9:**
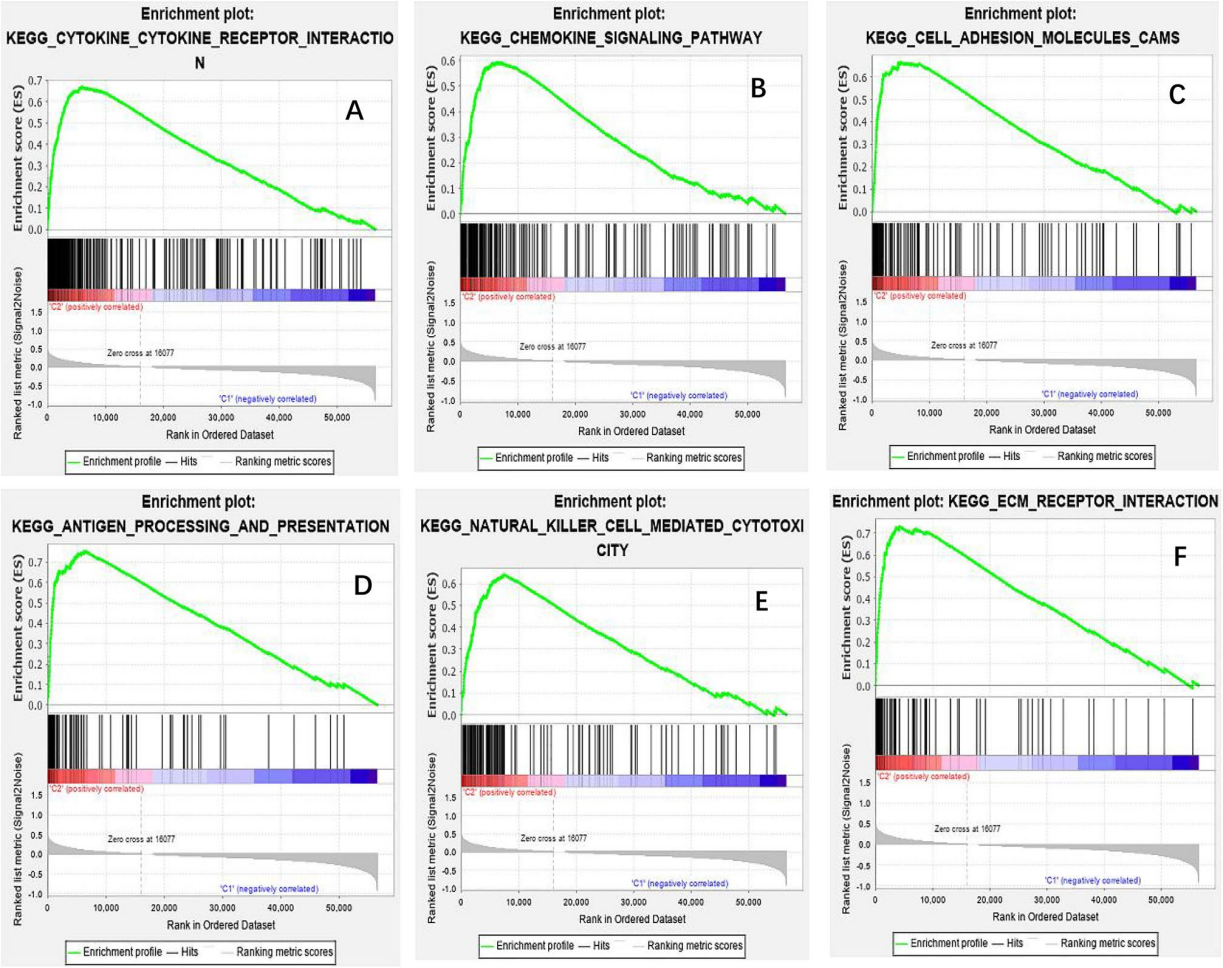
Part of the GSEA results are listed above. High risk of m6A-related lncRNAs were enriched in multiple cancer-related functions and pathways, which were upregulated in class C2. Both FDR q-value and FWER p-value < 0.01.

Differential analyses of immune cell infiltration and immune ological scores in distinct clusters were carried out to evaluate the effect of m6A-related lncRNAs on the TIME of BLCA (Table S4). The infiltration abundance of 22 types of immune cells in each cluster was shown in Fig. 7A. Additionally, cluster2 exhibits high levels of CD4 memory-activated T cells, macrophages M2, neutrophils and NK cells resting (p < 0.05; Fig. 7B–E), whereas cluster1 was more correlated with plasma cells (p < 0.05; Fig. 7F) and T cells regulatory (Tregs) (p < 0.001; Fig. 7A,G). As shown in Fig. 8A–C, All of the scores were higher in cluster2, indicating a higher level of immune-related cells in the tumor microenvironment (p < 0.05) (Table S5). We applied gene set enrichment analysis (GSEA) to explore the probable regulatory mechanisms underlying the variations in TIME between the two groupings. The hallmark pathways and functions involved in cluster2 included Chemokine Signaling Pathway, Cytokine-Cytokine Receptor Interaction, Cell Adhesion Molecules (CAMs), Natural Killer Cell Mediated Cytotoxicity, Antigen Processing and Presentation and ECM-Receptor Interaction (Fig. 9A–F). According to the results, the most enriched signaling pathway was “Chemokine signaling pathway”. These functions and pathways were up-regulated in cluster2, Both the FDR q-value and the FWER p-values were < 0.05. These functions and pathways might be implicated in the distinct TIME of cluster1/2.

### Construction of prognostic signatures based on m6A-related lncRNAs

**Figure 10:**
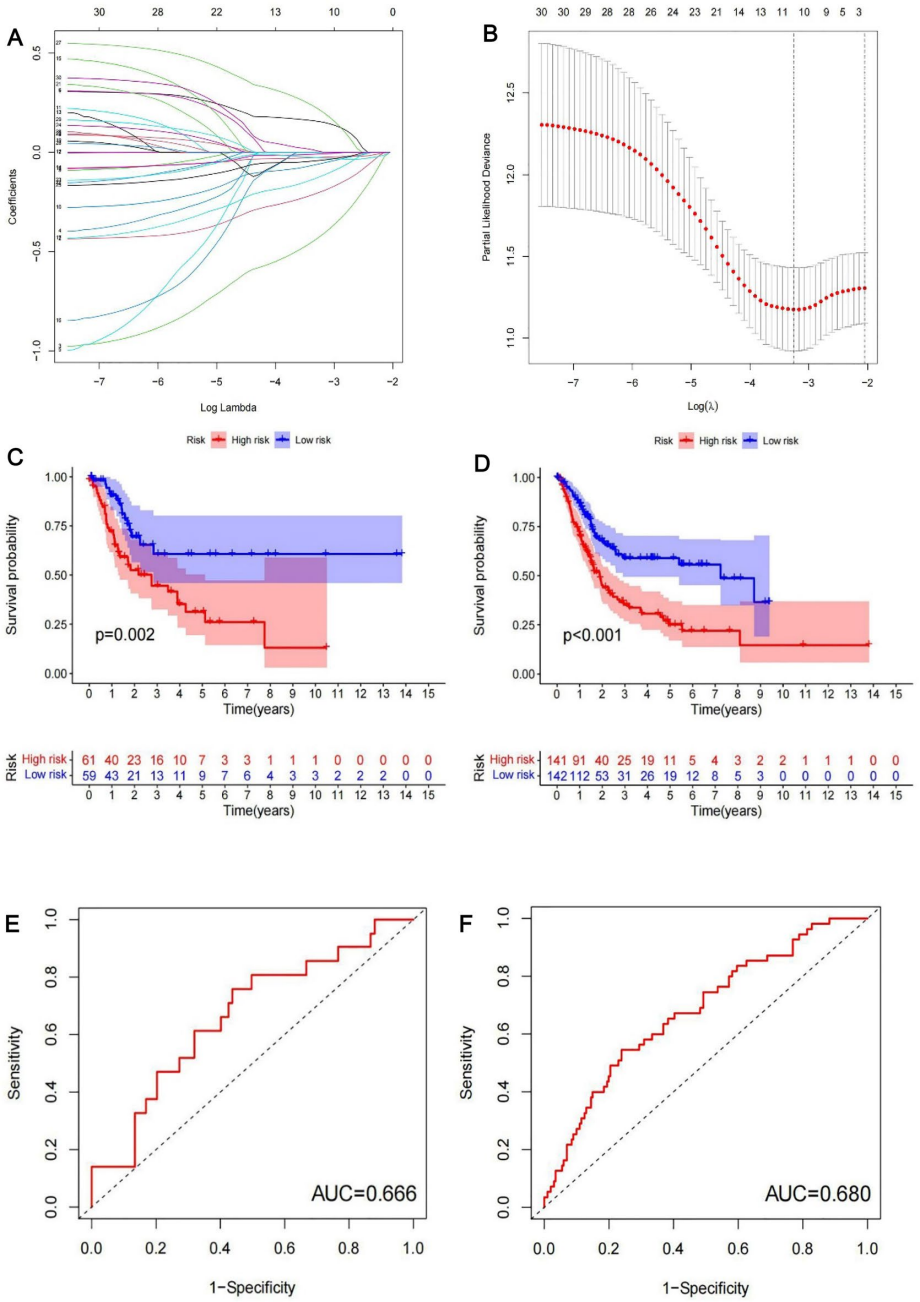
Construction of a novel prognostic risk signature for BLCA. (**A + B**) Lasso regression of 11 m6A-related prognostic lncRNAs was carried out to construct a prognostic model. (**C + D**) Overall survival analysis for patients in high/low risk ((**C**) represents test group, p < 0.01, while (**D**) represents train group, p < 0.001). (**E + F**) ROC curve to evaluate the accuracy of the predictive model ((**E**) represents test group, while (**F**) represents train group, AUC > 0.5).

**Figure 11:**
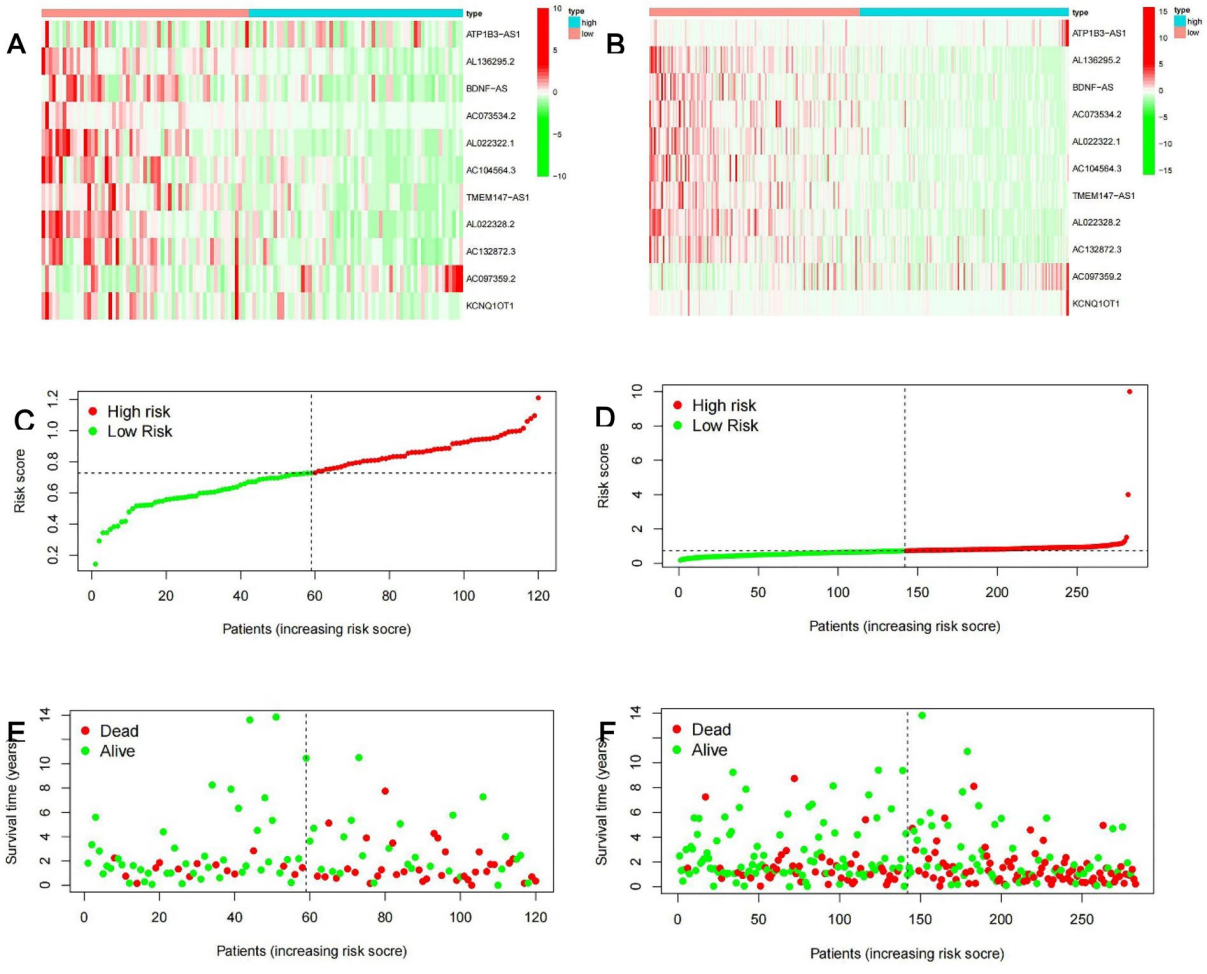
Distribution of risk score, OS, and OS status of the 11 prognostic m6A-related lncRNAs in the TCGA test cohort (**A + C + E**) and TCGA train cohort (**B + D + F**).

**Figure 12:**
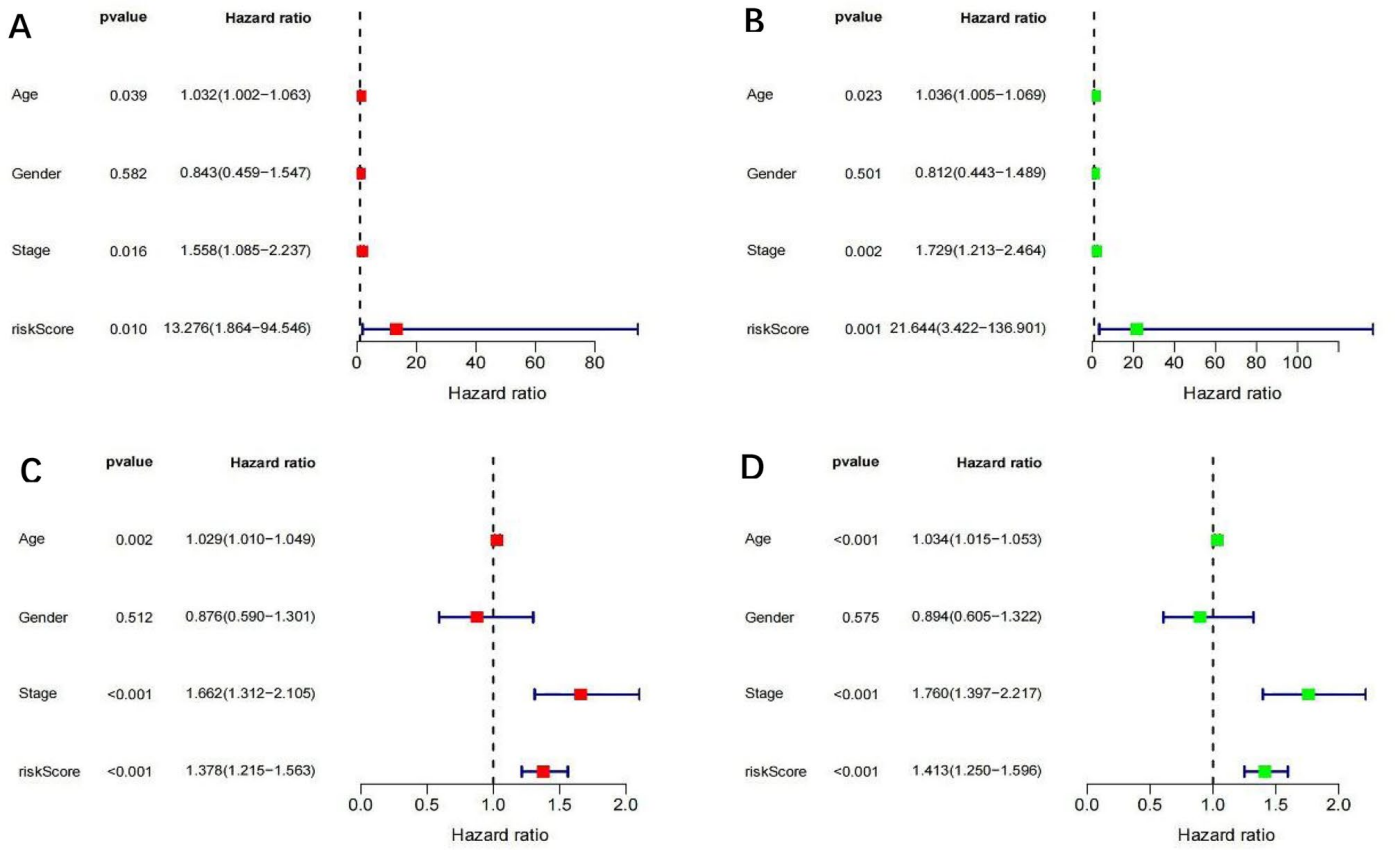
Multivariate and univariate analysis of independent prognostic analysis. ((**A + B**) represents test; (**C + D**) represents train, Stage, age and risk Score were risk factors for the prognosis of BLCA; (**A + C**) multivariate, (**B + D**) univariate analysis), p < 0.05.

**Figure 13:**
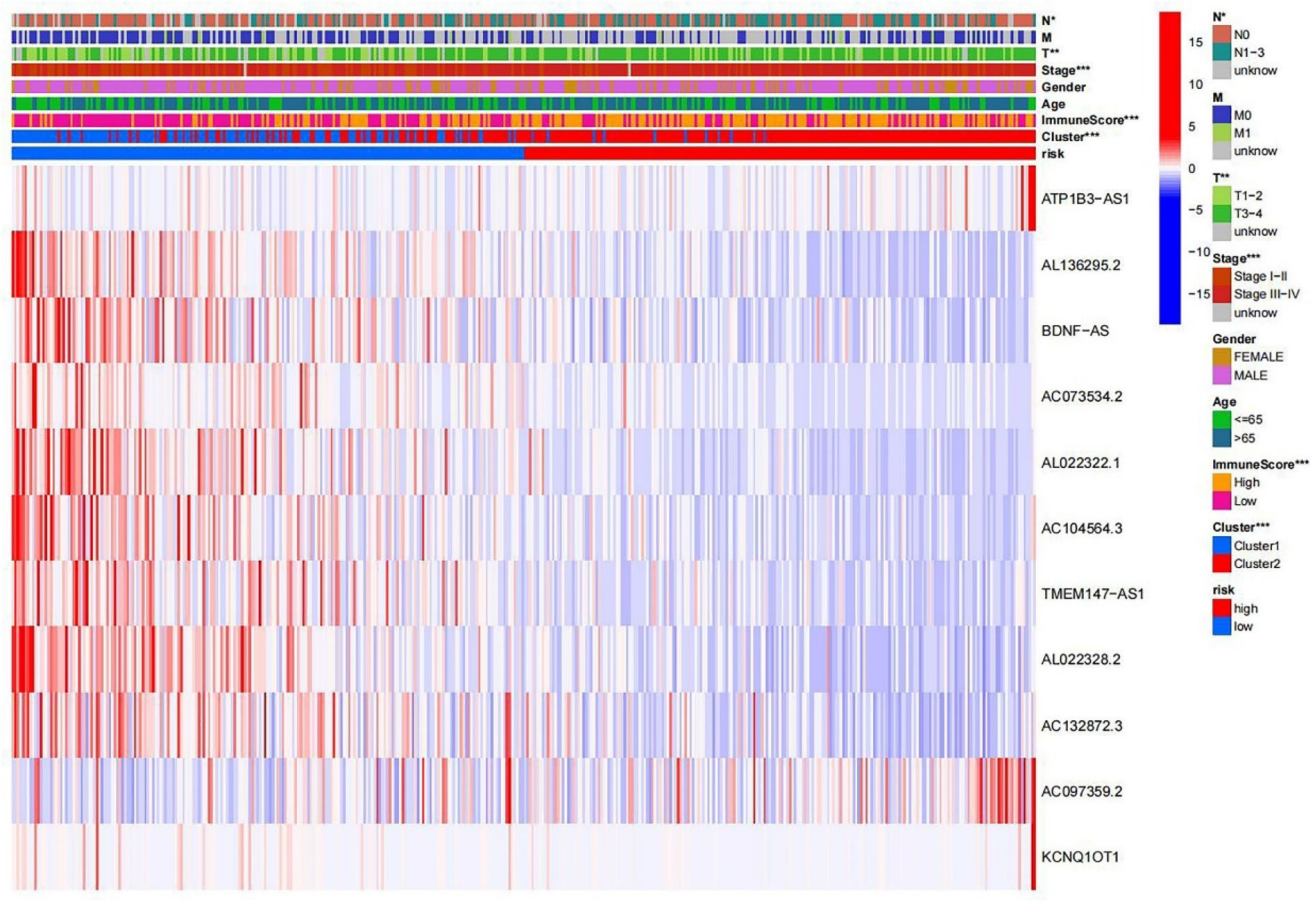
Heatmap and clinicopathologic features of high- and low-risk groups. *p < 0.05, **p < 0.01, and ***p < 0.001.

**Figure 14:**
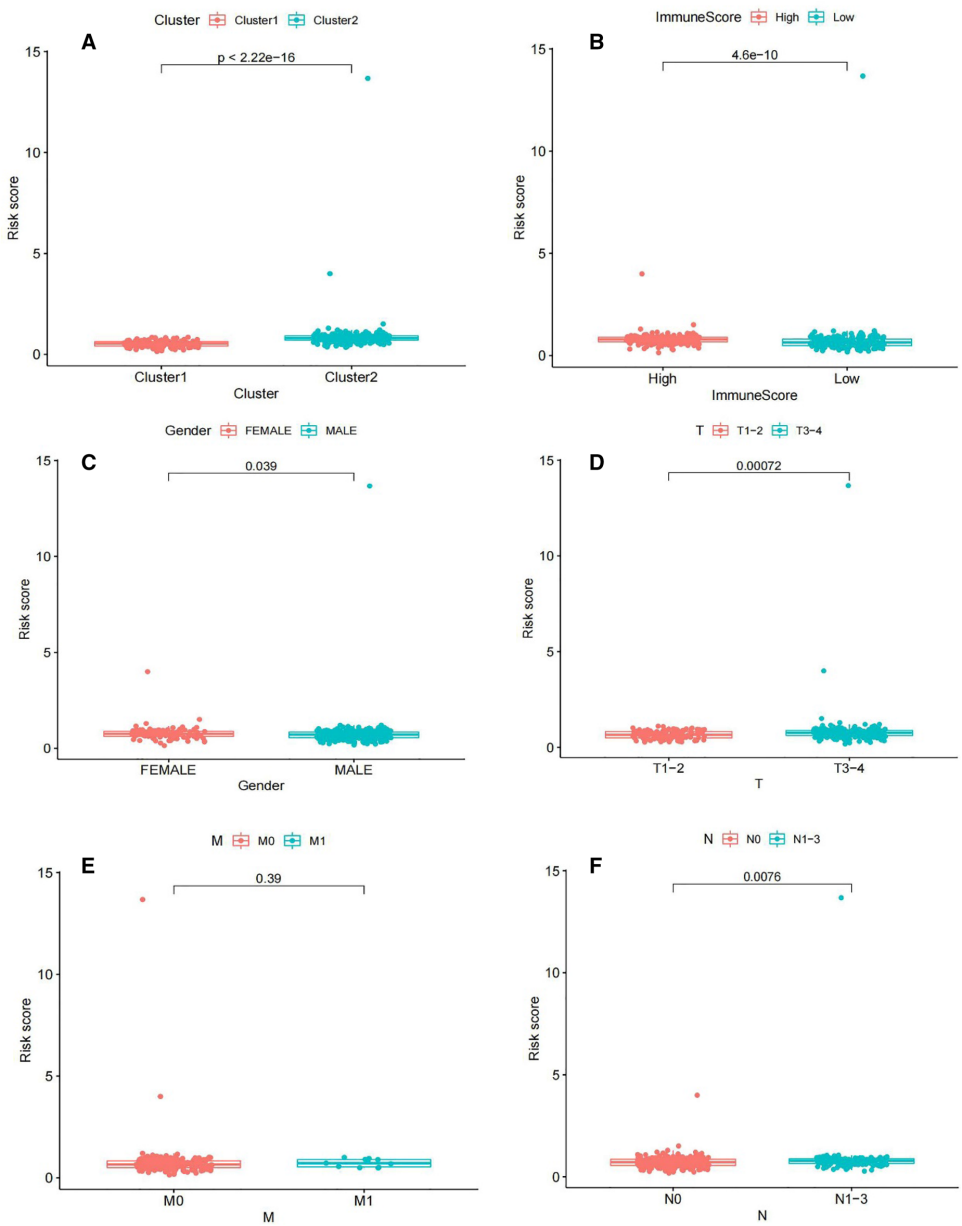
Box diagram of risk and clinical correlation analysis (subtype, gender, T stage and N stage were closely related to risk score, p < 0.05).

**Figure 15:**
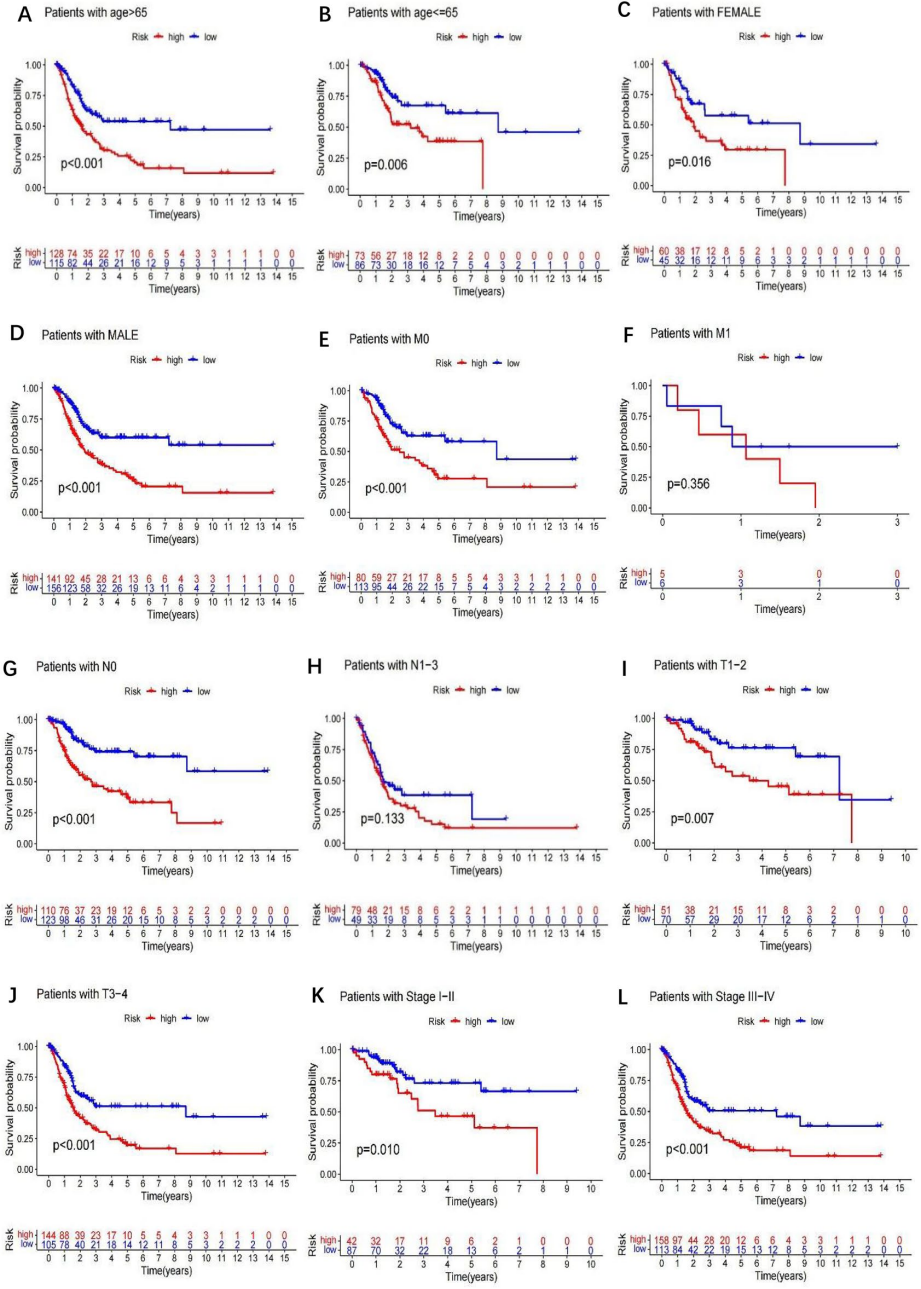
Survival curves for model validation. The model could be applied to different clinical groups: age (**A + B**), gender (**C + D**), M stage (**E + F**), N stage (**G + H**), T stage (**I + J**), and stage (**K + L**), (p < 0.05).

**Figure 16:**
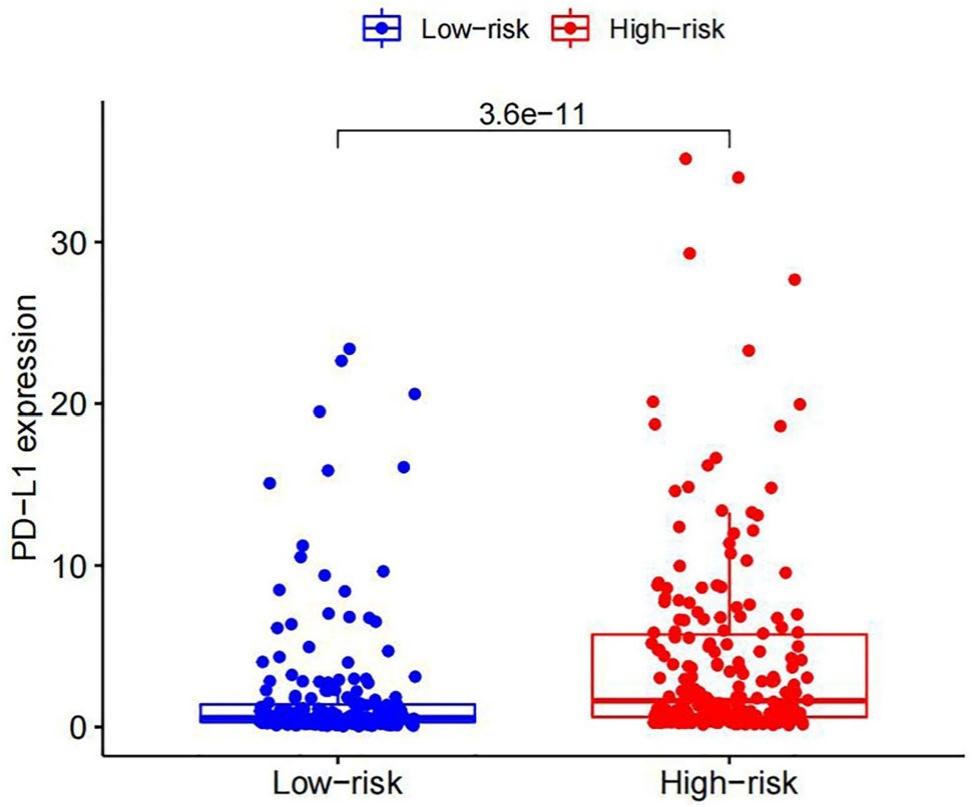
The PD-L1 expression level by risk score group. PD-L1 expression was higher in high-risk group (p < 0.001).

**Figure 17:**
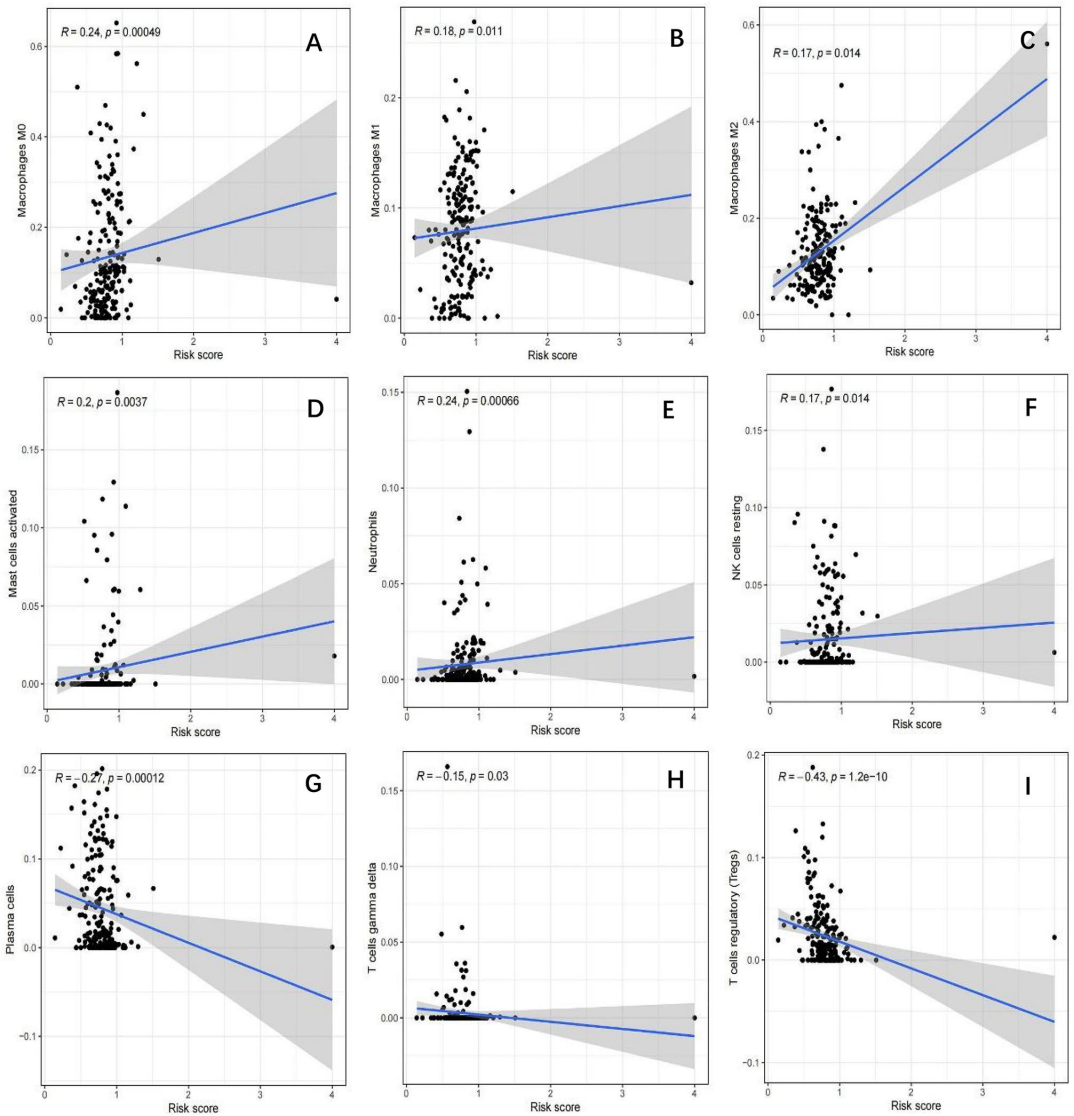
Relationships between the risk score and infiltration abundances of ten immune cell types. (**A**) Macrophages M0, (**B**) macrophages M1, (**C**) macrophages M2, (**D**) mast cells activated, (**E**) Neutrophils and (**F**) NK resting cells are positive related with risk score, R > 0 and p < 0.05. (**G**) Plasma cells, (**H**) T cells gamma delta and (**I**) T regulatory cells, R < 0 and p < 0.05.

A train group (70%) and a test group (30%) were utilized for the Kaplan–Meier survival analysis and associated plots to accurately predict the clinical outcome of m6A-related lncRNAs in BLCA patients, as shown in Fig. 10A,B, Tables S6 and S7. The results showed that the OS of patients in the high-risk group was significantly lower than that in the low-risk group, as shown in Fig. 10C,D. The corresponding ROC curve was acquired using the time-ROC package to test the accuracy of our model for predicting the survival of patients with the disease (Fig. 10E,F). ROC curve analysis showed that the AUC value of test group was 0.666, while the AUC value of train group was 0.680, indicating the considerable accuracy of our model for predicting the survival of patients with BLCA. Univariate and multivariate Cox analyses were performed to evaluate whether risk score was an independent prognostic factor for BLCA patients. The distribution of the risk scores, OS, OS status, and expression profiles of the eleven m6A-related lncRNAs in TCGA training and validation cohorts was displayed in Fig. 11A–F, which revealed that the higher the immune scores, the higher the risk. Both in the test group and train group, after univariate analysis obtained factors related to OS in BLCA patients, multivariate Cox regression analysis showed that risk score, stage and age were identified as independent prognostic factors (p < 0.01, HR > 1), in Fig. 12A–D. The link between risk score and clinical features, as well as cluster subgroups, was studied further. In the high-risk and low-risk groups, the heatmap showed the expression levels of eleven m6A-related lncRNAs (Fig. 13). AL136295.2, BDNF–AS, AC073575. 4, AC074117. 1, AC073534. 2, AL022322. 1, AC004076. 2, AC104564. 3, TMEM147–AS1, AC104532. 2, AC116914. 2 were highly expressed in cluster1 and low risk group, while ATP1B3–AS1 and AC097359. 2 were highly expressed in cluster2 and high-risk group. Furthermore, the different risk scores among subtype (p < 0.001), immune scores (p < 0.001), stage (p < 0.001) and N stage (p < 0.05) were examined in this heatmap. The results show that the risk score of cluster2, higher immune scores, stage III–IV and N1–N3 is significantly higher. These findings indicate that the risk score of BLCA patients may profoundly influence clinical outcomes. We further validate the relationship between risk scores and subtypes, immune scores and TMN stage (Fig. 14A–F). The results showed that the risk score of cluster2, T3–4 and N1–3 were significantly higher than that of cluster1. Although there is no significant difference in risk score between high and low immunity scores, it can be seen that the median risk score of high immunity score is higher than that of low immunity score. To evaluate and verify if our model could be applied to diverse clinical parameters, we utilized model validation for clinical groups, as shown in Fig. 15. The result indicated that our model could be applied to the following different clinical parameters: age, gender, lymph node metastasis, stage and T stage (p < 0.05). In our BLCA model, genetic differential analysis was used to examine the expression differences of target genes in the different risk groups, as shown in Fig. 16. The expression level of PD-L1 was higher in high-risk group (p < 0.001). The influence of eleven m6A-related lncRNAs on the BLCA immune microenvironment was estimated using the relationship between the risk score and immune infiltration levels (Fig. 17). The risk score was positively correlated with the infiltration levels of Macrophages M0, Macrophages M1, Macrophages M2, Mast cells activated, Neutrophils and NK resting cells with R > 0 and p < 0.05. A significantly negative correlation was observed between the risk score and infiltration levels of Plasma cells, T cells gamma delta and T cells regulatory (Tregs) with R < 0 and p < 0.05. Risk signatures based on m6A-related lncRNAs are likely to have a role in BLCA's immune microenvironment regulation.

## Discussion

If not treated effectively, BLCA is a complicated disease with high morbidity and fatality rates^35^. M6A modification regulates mRNA splicing, stability, nuclear export, and translation^36^. Furthermore, The alteration of m6A has an impact on lncRNA processing, as well as the processes that control cell proliferation and maturation^37^. LncRNA-based signatures have been shown to accurately predict BLCA survival and recurrence^38,39^. For instance, NKILA lncRNA boots tumor immune evasion by sensitizing T cells to activation-induced cell death^40^. Several studies have found that using m6A-related lncRNAs to build prognostic models was effective in predicting tumor prognosis^43–45^. In the current study, co-expression analysis was carried out to identify the correlation between m6A-related gene expression and lncRNAs. This was followed by univariate Cox regression analysis for the identification of m6A-related prognostic lncRNAs. As a result, 30 m6A-associated lncRNAs were found to be strongly related to overall survival outcomes. Some m6A-related lncRNAs are highly expressed in tumor tissues, while others are highly expressed in normal tissues (p < 0.05). Therefore, the expression, prognostic value, and immune significance of m6A-related lncRNAs in BLCA were researched which could guide future research.

Next, two molecular subtypes (clusters 1/2) were identified by consensus clustering of 30 m6A-related lncRNAs. The prognosis and different clinicopathological aspects of BLCA were determined by the cluster1/2 subtype, which was directly associated to PD-L1, immunological scores, and immune cell infiltration levels. Patients in cluster2 showed a worse survival rate than those in cluster1, which could be due to the greater importance of immunological scores and PD-L1 expression levels in cluster2. These findings backed up the findings of a previous study, which found that bladder cancer patients with high immunological and stromal scores had a lower overall survival rate^41^. We also discovered that PD-L1 expression was considerably higher in cluster2 than in cluster1. In addition, it has been observed that PD-L1 is significantly negatively correlated with LINC0260, AC104532. 2, AL022328.2 and EHMT2–AS1. Further research is required to see whether the regulators can estimate the effectiveness of immunotherapy in BLCA patients. Increasing evidence also suggests that targeting lncRNA UCA1 with CRISPR-Cas9, which blocks PD-1 function, can improve antitumor activity in BLCA patients^42^. However, research on m6A alteration of lncRNAs is currently small in number. Thus, there is an urgent need for further research on lncRNA m6A modification and recognition corroborate our findings.

Moreover, there was a substantial variation in TIME between cluster1/2 subtypes. The results revealed that CD4 memory-activated T cells, macrophages M2, neutrophils, and NK cells resting in cluster2 were greater than those resting in cluster1, but plasma cells and T cells regulatory (Tregs) were strongly clustered in cluster1. Furthermore, in agreement with our findings, tumor-associated macrophages are critical immunosuppressive cells driving tumorigenesis and metastasis^43,44^. Macrophages that infiltrate the tumor microenvironment are usually referred to as tumor-associated macrophages (TAMs)^45^. TAMs have been shown to promote tumor cell proliferation, invasion, and metastasis, strengthen angiogenesis, and suppress the anti-tumor response^46^. It has been observed that BLCA patients with larger numbers of macrophages, particularly macrophages M2, had poorer prognoses^47,48^. Next, we carried out GSEA. “CHEMOKINE SIGNALING PATHWAY” was the most significantly enriched signaling pathway. CCL2, CCL3, CCL4, and CCL5 are CC chemokines that are well-known chemotactic elements for macrophage populations in tumors^49,50^. CCL2 stimulation shifts human peripheral blood CD11b + cells toward a CD206 + M2-polarized phenotype according to Roca et al.^51^. Tripathi et al. also discovered that oncostatin M and the chemokine CCL11/eotaxin, both generated by hypoxic cancer cells, shifted macrophages toward an M2 phenotype^52,53^. Additionally, CCL17 and CCL22 can promote Treg migration by interacting with the CCR4 receptor^54^. Thus, attraction of immunosuppressive immune cells through chemokine production is one of the pro-tumoral characteristics of TAMs. Taken together, our findings demonstrate that m6A methylation of lncRNA plays a crucial role in shaping the TIME for immune evasion, providing novel insights for effective cancer immunotherapy.

Lasso regression was used to establish a statistical model for m6A-related lncRNAs. The OS of the patients in high-risk group was shorter than that of the patients in low-risk group in the TCGA test and train cohorts. Cluster2 had considerably higher risk ratings, immunological scores, and PD-L1 levels, according to further research. This observation was consistent with previous results. previous research have found that PD-L1 can bind to PD-1 on T cells, B cells, and macrophages activated on the surface of tumor cells, thus showing immunosuppressive effects^55^. A high level of PD-L1 expression has been linked to malignancy and a poor prognosis in BLCA patients, and such individuals have a greater rate of recurrence following surgery^56–58^. The results further confirming that the PD-L1 gene may be an oncogene of bladder cancer. Furthermore, this conducted model could be applied to different clinical groups. The risk score was found to be an independent prognostic factor for BLCA patients in both univariate and multivariate Cox regression models. Further experimental studies is necessary to determine the regulation of these lncRNAs by m6A modification and to clarify the associated processes in bladder cancer progression and immune evasion. Studies with larger sample sizes are also needed to confirm the prognostic value of m6A-related lncRNA risk score.

## Conclusion

In conclusion, this study thoroughly identified the expression of m6A-related lncRNAs in BLCA, their correlation with PD-L1, effects on the TIME and potential regulatory mechanisms. Two BLCA subtypes (cluster1/2) were identified, each of which classified the prognosis of BLCA patients while also presenting considerably distinct TIME. By modifying TIME and PD-L1 expression, m6A-related lncRNAs may promote the responsiveness of BLCA patients to immunotherapy. GSEA further identified that the m6A-related lncRNAs might be involved in the regulation of BLCA immune microenvironment in synergy with the “CHEMOKINE SIGNALING PATHWAY”. More importantly, we developed and validated an prognostic model based on m6A-related lncRNA risk score with robust prognostic value and the ability to predict response to immunotherapy in patients with bladder cancer. The risk score is highly correlated with the malignant clinicopathological features of BLCA, which enhanced the understanding of m6A-related lncRNAs in TIME cells infiltration and immune evasion, providing novel insights for guiding more effective immunotherapy strategies in BLCA.

## Methods

### Ethics statement

Genes were investigated exclusively using sequences available in public databases. This research did not include human or animal subjects. The all study were performed in accordance with the relevant guidelines and regulations and adhered to the Declaration of Helsinki.

### Sample data acquisition and collation

Transcriptome RNA sequencing data of 433 colorectal cancer samples were gained from the TCGA website (https://portal.gdc.cancer.gov/)^59^, which contains 414 tumor samples and 19 adjacent normal samples. Then, utilising PERL software (https://www.perl.org/), we created the mRNA matrix and used the associated script to organize transcriptomic data and transform gene IDs. In addition, the authors collected the clinical information of 409 patients with bladder cancer from the TCGA website, including futime, fustat, age, gender, stage and tumor staging. And, the clinical statistical analyses were performed using the same software and a specific script.

### Identification of m6A related lncRNAs

To distinguish mRNAs and lncRNAs by employing the collated transcriptome data, we stablished a gene expression matrix and human configuration file including the expression levels of related gene profiles. Then, the gene IDs were then converted to gene names using data from the Ensembl database (http://asia.ensembl.org/info/data/index.html). By running biotype.pl script, we got m6A gene expression file and LncRNA expression file, and co-expression analysis was carried out to illustrate the connection between m6A-related gene expression. Furthermore, the “BiocManager” ackage was used to combine lncRNA expression data and clinical survival data to acquire prognostic m6A-related lncRNAs, and a network plot was depicted via the igraph package to visualize the correlation. The survival package estimated the confidence interval and hazard ratio, and then forest plots were used to visualize the results of the univariate Cox regression analysis. The differential expression data of prognosis-related m6A-related lncRNAs between tumor and normal tissues were acquired through the limma package, pheatmap package, reshape2 package and ggpubr package in R software. Differences with p < 0.05 were deemed statistically meaningful. To visualize the differences of expression, heatmaps and boxplots were drafted.

### The role of m6A-related lncRNAs

We used the “ConsensusClusterPlus” tool to examine the biological features of m6A-related lncRNAs in BLCA (http://www.bioconductor.org/, 1000 iterations and resampling rate of 80%) to categorize BLCA patients from the TCGA database into two groups. Moreover, survminer and survival programs were used to do a survival analysis based on lncRNA subtypes. A heatmap was depicted to demonstrate the correlation between lncRNAs and clinicopathological indicators. the standard names of the target genes was identified via NCBI (https://www.ncbi.nlm.nih.gov/). Differences with p < 0.05 were deemed statistically meaningful.

### The role of immune cells infiltration and the tumor microenvironment

CIBERSORT (http://cibersort.stanford.edu/), a deconvolution algorithm based on gene expression was computed the composition of immune cells^60^. Based on the expression profile data of BLCA in the TCGA database, the CIBERSORT software was used to calculate the infiltration level of 22 immune cells. Subsequently, was used the ESTIMATE algorithm to calculate the immune scores of each patient, and the different immune scores between the two cluster subgroups was evaluated^61^. The biological processes involved in distinct subgroups were studied using gene set enrichment analysis (GSEA). GSEA hallmarks were used to identify predetermined gene sets, and p-values were determined via 5000 permutations according to the gene set. As referenced in the Results section, a pathway or function with a p-value < 0.05 and a false discovery rate (FDR) < 0.05 was considered relevant.

### m6A-related lncRNAs prognostic model

First, we used the createDataPartition function to randomly divide the sample into two groups, a train group (70%) and a test group (30%), and the data of the training group and test group from TCGA. The prognostic signature of m6A-related lncRNAs were identified by Lasso regression analysis to build a m6A-related lncRNAs Prognostic Model. The following computational equation was utilized to calculate the coefficients for each bladder cancer case: risk score = sum of coefficients × the lncRNA expression. In both the training and test cohorts, the risk score of all BlCA patients was calculated. The patients were then divided into high-and low-risk groups, with the median value of the risk score serving as the cutoff point. Then the survival curves of different groups were depicted, in which high-risk and low-risk groups were compared. To evaluate the accuracy of our model for predicting the survival of patients with the disease, a corresponding ROC curve was obtained via the time ROC package. Multivariate and univariate analyses were carried out to evaluate whether our model was independent of other clinical prognostic factors that affect patient outcomes. In addition, model validation for clinical groups was utilized to test and verify whether our model could be applied to different clinical groups. A heatmap and survival curves were generated to clarify related high-risk and low-risk m6A-related lncRNAs and expound the correlation of clinical characteristics and our prognostic risk model. Genetic differential analysis was performed to assess the expression difference of target genes in different risk groups in our model in bladder cancer. Moreover, the correlation between the risk score and the abundance of immune cells was also calculated to visualize this nexus and assess whether immune cells were beneficial or detrimental.

## Supplementary Information

Supplementary Information 1.

Supplementary Information 2.

Supplementary Information 3.

Supplementary Information 4.

Supplementary Information 5.

Supplementary Tables.

## Data Availability

Publicly available datasets were analyzed in this study. This data can be found here: TCGA database (http://www.cancer.gov/tcga). The raw data and relative software/code can be accessed in a Supplementary File. The data generated during the current study are available from the corresponding author on reasonable request.
